# Effects of Hall Current and Viscous Dissipation on Bioconvection Transport of Nanofluid over a Rotating Disk with Motile Microorganisms

**DOI:** 10.3390/nano12224027

**Published:** 2022-11-16

**Authors:** Abdullah K. Alzahrani

**Affiliations:** Mathematical Modelling and Applied Computation (MMAC) Research Group, Department of Mathematics, Faculty of Science, King Abdulaziz University, P.O. Box 80203, Jeddah 21589, Saudi Arabia; akalzahrani@kau.edu.sa

**Keywords:** nanofluid, motile microorganisms, rotating disk, Hall current, viscous dissipation, partial differential equations (PDEs)

## Abstract

The study of rotating-disk heat-flow problems is relevant to computer storage devices, rotating machineries, heat-storage devices, MHD rotators, lubrication, and food-processing devices. Therefore, this study investigated the effects of a Hall current and motile microorganisms on nanofluid flow generated by the spinning of a disk under multiple slip and thermal radiation conditions. The Buongiorno model of a nonhomogeneous nanofluid under Brownian diffusion and thermophoresis was applied. Using the Taylor series, the effect of Resseland radiation was linearized and included in the energy equation. By implementing the appropriate transformations, the governing partial differential equations (PDEs) were simplified into a two-point ordinary boundary value problem. The classical Runge–Kutta dependent shooting method was used to find the numerical solutions, which were validated using the data available in the literature. The velocity, motile microorganism distribution, temperature, and concentration of nanoparticles were plotted and comprehensively analyzed. Moreover, the density number, Sherwood number, shear stresses, and Nusselt number were calculated. The radial and tangential velocity declined with varying values of magnetic numbers, while the concentration of nanoparticles, motile microorganism distribution, and temperature increased. There was a significant reduction in heat transfer, velocities, and motile microorganism distribution under the multiple slip conditions. The Hall current magnified the velocities and reduced the heat transfer. Thermal radiation improved the Nusselt number, while the thermal slip conditions reduced the Nusselt number.

## 1. Introduction

The heat transport process is imperative in chemical, mechanical, engineering, and biomedical systems. The performance of devices such as heat exchangers, gas turbine blades, combustors, microelectronic boards, and solar panels depends on the rate of heat transport and can be modulated by altering the heat transfer coefficient and improving the thermal exchange properties of the working fluids such as oil, ethylene glycol, and water. The use of nanofluids represents a decisive solution to extemporizing the heat transport process. The applications of nanofluids in numerous disciplines were described by Abu-Nada [1]. Choi and Eastman [2] confirmed the efficacy of this alternative heat transport fluid in various applications by establishing the improved heat transport properties of nanofluids. Thus far, in the field of nanofluidics research, a great number of theoretical studies have been conducted by applied mathematicians, due to the predominant applications such as electronic device cooling, oil recovery systems, solar energy systems, nuclear systems cooling, and renewable energies. Buongiorno [3] established that the non-uniform relative velocity between the nanoparticles and the base fluid plays a role in the advancement of heat transfer in nanofluids through the Brownian diffusion of the nanoparticles. Nield and Kuznetsov [4] examined the effects of nanofluidics on boundary layer transfer with natural convection using a similarity approach. Kuznetsov and Nield [5] re-examined the Cheng–Minkowycz problem related to the Buongiorno model and found that the diffusion generated by the zig-zag movement of nanoparticles causes the development of a heat field. Wakif et al. [6] investigated the Buongiorno hybrid nanofluidic transition carrying solid alumina particles over an extended plate. Rasheed et al. [7] calculated the numerical solution for the 3D flow of nanofluids under rotation and convection conditions. Gumber et al. [8] studied a submerged nanofluid with micropolar particles subjected to Rosseland radiation with transpiration cooling on a vertical plate. Mahanthesh [9] examined the role of nanofluid agglomeration in transport behavior subject to nonlinear radiation and convection conditions. Areekara et al. [10] used the Buongiorno nanofluid model to study the transition of a nanofluid subject to zero-mass flow conditions and highlighted the biomedical applications of the nanofluid. For recent research on nanofluids under various physical conditions, refer to [11,12,13,14,15].

The upward movement of mobile microorganisms generates bioconvection in a fluid. The swimming of mobile microorganisms (e.g., algae and bacteria) in a base fluid makes the fluid denser but prevents the agglomeration of nanoparticles and allows the nanofluid to effectively improve its heat transport characteristics. Bioconvection is useful in a variety of biotechnology applications, including biosensors, biopolymer fabrication, drug delivery, microsystems, and the recovery of microbial oils. Kuznetsov [16] investigated the consequences of oxytactic microorganisms swimming in a nanofluid layer and found that microbes speed up the onset of convection. Sampath et al. [17] analyzed a convective heated and submerged nanofluid with gyrotactic microorganisms and concluded that due to the bioconvection Lewis number, the heat transfer rate increased by 37.3%. Chu et al. [18] examined the effects of gyrotactic microorganisms on a Maxwell nanofluid under Cattaneo–Christov heat flow on a nanomaterial surface. Ayodeji et al. [19] studied a magnetic nanofluid with bioconvection on a stretched plate under the conditions of slip and Brownian motion. They determined that Brownian diffusion and the Peclet number increased the temperature of the nanofluid. Khan et al. [20] predicted the bioconvection of a magnetic nanofluid on a higher paraboloid surface. Shehzad et al. [21] analyzed Cattaneo–Christov heat and mass fluxes in the bioconvection transport of micropolar nanofluid. Khan et al. [22] examined thixotropic nanofluid flow with gyrotactic microorganisms and activation energy. Ferdows et al. [23] reported bioconvection over an exponentially extended plate using magneto-nanofluids. Waqas et al. [24] analyzed the effects of second-order slip conditions, chemical reactions, and thermal radiation on bioconvection in magneto-Carreau–Yasuda nanofluids. Recently, Muhammad et al. [25] observed bioconvection in a thixotropic magneto-nanofluid. However, the phenomenon of bioconvection in rotating geometries under slip conditions has yet to be explored.

The traditional problem of the rotating disk was studied by Von Karman [26], who introduced transformations to generate self-similar equations. Flow simulations on a spinning disk provide the information required for various applications, such as spinning machines, computer disk drives, turbine systems, crystal growth processes, spinning viscometers, and food processing. Benton [27] discussed the applications of spinning-disk problems. Turkyilmazoglu [28] considered porous-disk flow with a Hall current and highlighted the consequences and applications of Hall currents. Sheikholeslami [29] investigated a magnetic nanofluid on a rotating disk; similarly, Turkyilmazoglu [30] revealed the heat transfer patterns of the nanofluid under the revolution of the disk. Hayat et al. [31] extended this study [30] to examine the partial slip effects and found that they reduced the velocity and thus accelerated the shear stress on the disk surface. Abdel-Wahed and Akl [32] analyzed the effects of a Hall current on a magneto nanofluid on a rotating disk and reported that the Hall current improved the heat transport on the disk surface. Hayat et al. [33] studied the biphasic flow of a nanofluid on a disk under sliding conditions and described the characteristics of the thermophoresis in the heat field. Rehman et al. [34] extended this study [33] by considering a Casson nanofluid. Recent studies on the Hall current and rotating disks can be found in [35,36,37,38,39,40,41,42].

The literature review revealed that only a few studies have explored the effect of Hall currents and viscous dissipation on nanofluid flow with gyrotactic microorganisms on a spinning disk. The additional features of multiple slip conditions and thermal radiation with viscous heating in the proposed mathematical problem distinguish this novel study from others in the literature. The consideration of Buongiorno’s biphasic model and viscous heating with bioconvection resulted in a highly non-linear model that was solved by the shooting method. The density number, Sherwood number, shear stress, and Nusselt number were calculated and analyzed. The paper is organized as follows: Section 2 deals with the formulation of the model; Section 3 covers the shooting solution of the self-similar equations; the findings are presented in detail in Section 4; and the conclusions are provided in Section 5.

## 2. Formulation of the Problem

Let us consider the three-dimensional flow of a nanofluid submerged with gyrotactic microorganisms on an infinite revolving disk. The nanofluid of a constant density (ρ), thermal conductivity (k), specific heat ($\rho C_{p}$), electrical conductivity (σ), Brownian motion coefficient ($D_{B}$), thermophoresis coefficient ($D_{T}$), microorganism diffusivity ($D_{W}$), and dynamic viscosity (μ) obeys the non-homogeneous model of Buongiorno. The Hall effect with a strong magnetic field is applied to the flow system. The generalized mathematical problem, which describes the laminar flow, is seen below:

(1) $$\nabla \cdot \vec{U}=0$$

(2) $$\rho \left(\frac{\partial \vec{U}}{\partial t}+\left(\vec{U}\cdot \nabla \right)\vec{U}\right)=-\nabla p+\mu \nabla^{2}\vec{U}+\vec{J}\times \vec{B}$$

(3) $$\rho C_{p}\left(\frac{\partial T}{\partial t}+\left(\vec{U}\cdot \nabla \right)T\right)=k\nabla^{2}T+\Phi +{\left(\rho C_{p}\right)}_{np}\left(D_{B}\nabla C\cdot \nabla T+\frac{D_{T}}{T_{\infty }}\nabla T\cdot \nabla T\right)-\nabla q_{r},$$

(4) $$\frac{\partial C}{\partial t}+\left(\vec{U}\cdot \nabla \right)C=D_{B}\nabla^{2}C+\frac{D_{T}}{T_{\infty }}\nabla^{2}T,$$

(5) $$\frac{\partial W}{\partial t}+\nabla \cdot \left(WU+W\gamma -D_{W}\nabla W\right)=0,$$

where γ is defined as

(6) $$\gamma =\left(\frac{bW_{c}}{C_{w}-C_{\infty }}\right)\nabla C.$$

The Ohms law with no electric field to define the Hall current is

(7) $$\vec{J}=\sigma \left(\vec{U}\times \vec{B}-m\left(\vec{J}\times \vec{B}\right)\right)$$

The radiative heat flux $q_{r}$ is defined as

(8) $$q_{r}=-\frac{4\sigma^{*}}{3k^{*}}\nabla T^{4}$$

The operators in the cylindrical system are

(9) $$\left(\nabla ,\nabla^{2}\right)=\left(\frac{\partial }{\partial r}\hat{e_{r}}+\frac{1}{r}\frac{\partial }{\partial \varphi }\hat{e_{\varphi }}+\frac{\partial }{\partial z}\hat{e_{z}},\;\;\frac{\partial^{2}}{\partial r^{2}}+\frac{1}{r}\frac{\partial }{\partial r}+\frac{1}{r^{2}}\frac{\partial^{2}}{\partial \varphi^{2}}+\frac{\partial^{2}}{\partial z^{2}}\right),$$

where t is the time, $\vec{U}$ is the velocity vector, $\vec{J}$ is the current density, $\vec{B}$ is the magnetic field, p is the pressure, T is the temperature, C is the nanoparticle volume fraction, W is the density of microorganisms, ${\left(\rho C_{p}\right)}_{np}$ is the specific heat of the nanoparticles, Φ is the viscous dissipation, $T_{\infty }$ is the ambient temperature, $m=1/n_{e}n$ is the Hall factor, $n_{e}$ is the electron concentration, n is the electron charge, b is the chemotaxis constant, $W_{c}$ is the extreme speed of cell swimming, $k^{*}$ is the Rosseland mean absorption, $\sigma^{*}$ is the Stefan–Boltzmann constant, and ΔC is the concentration gradient.

**Figure 1:**
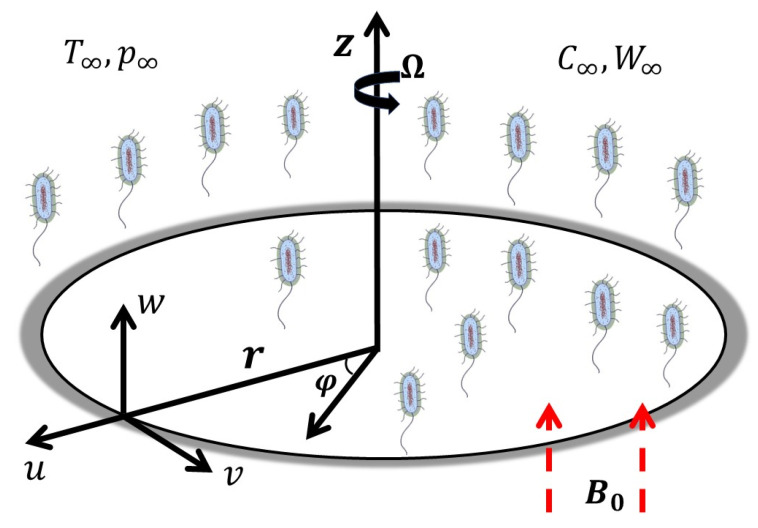
Physical configuration of the problem.

As seen in the geometric diagram (Figure 1), the disk rotates with a velocity Ω around the z-axis. Considering a cylindrical system (r, φ,z), the r-axis is constrained along the radial direction, and the z-axis is taken as normal to it. The disk surface has a uniform temperature, nanoparticle concentration, and density of microorganisms, which are constant under ambient conditions. A steady flow is considered, without suction or injection. The disk surface is subjected to multiple slippage conditions. The simplified component equations are as follows (see [30,32,36]):

(10) $$\frac{\partial u}{\partial r}+\frac{v}{r}+\frac{\partial w}{\partial z}=0,$$

(11) $$\rho \left(u\frac{\partial u}{\partial r}+w\frac{\partial u}{\partial z\;}-\frac{v^{2}}{r}\right)=-\frac{\partial p}{\partial r}+\mu \left(\frac{\partial^{2}u}{\partial r^{2}}+\frac{1}{r}\frac{\partial u}{\partial r}-\frac{u}{r^{2}}+\frac{\partial^{2}u}{\partial z^{2}}\right)-\frac{\sigma B_{0}^{2}}{1+m^{2}}\left(u-mv\right),$$

(12) $$\rho \left(u\frac{\partial v}{\partial r}+w\frac{\partial v}{\partial z\;}+\frac{uv}{r}\right)=\mu \left(\frac{\partial^{2}v}{\partial r^{2}}+\frac{1}{r}\frac{\partial v}{\partial r}-\frac{v}{r^{2}}+\frac{\partial^{2}v}{\partial z^{2}}\right)-\frac{\sigma B_{0}^{2}}{1+m^{2}}\left(v+mu\right),$$

(13) $$\rho \left(u\frac{\partial w}{\partial r}+w\frac{\partial w}{\partial z}\right)=-\frac{\partial p}{\partial z}+\mu \left(\frac{\partial^{2}w}{\partial r^{2}}+\frac{1}{r}\frac{\partial w}{\partial r}+\frac{\partial^{2}w}{\partial z^{2}}\right),$$

$$\rho C_{p}\left(u\frac{\partial T}{\partial r}+w\frac{\partial T}{\partial z}\right)=\left(k+\frac{16\sigma^{*}T_{\infty }^{3}}{3k^{*}}\right)\left(\frac{\partial^{2}T}{\partial r^{2}}+\frac{1}{r}\frac{\partial T}{\partial r\;}+\frac{\partial^{2}T}{\partial z^{2}}\right)+D_{B}{\left(\rho C_{p}\right)}_{np}\left(\frac{\partial T}{\partial z}\frac{\partial C}{\partial z}+\frac{\partial T}{\partial r}\frac{\partial C}{\partial r}\right)$$

$$+\frac{D_{T}{\left(\rho C_{p}\right)}_{np}}{T_{\infty }}\left({\left(\frac{\partial T}{\partial z}\right)}^{2}+{\left(\frac{\partial T}{\partial r}\right)}^{2}\right)+\mu \left({\left(\frac{\partial u}{\partial z}+\frac{\partial w}{\partial r}\right)}^{2}+{\left(\frac{\partial v}{\partial r}-\frac{v}{r}\right)}^{2}\right)$$

(14) $$+2\mu \left({\left(\frac{\partial u}{\partial r}\right)}^{2}+{\left(\frac{u}{r}\right)}^{2}+{\left(\frac{\partial w}{\partial z}\right)}^{2}\right),$$

(15) $$u\frac{\partial C}{\partial r}+w\frac{\partial C}{\partial z}=D_{B}\left(\frac{\partial^{2}C}{\partial r^{2}}+\frac{1}{r}\frac{\partial C}{\partial r\;}+\frac{\partial^{2}C}{\partial z^{2}}\right)+\frac{D_{T}}{T_{\infty }}\left(\frac{\partial^{2}T}{\partial r^{2}}+\frac{1}{r}\frac{\partial T}{\partial r\;}+\frac{\partial^{2}T}{\partial z^{2}}\right),$$

(16) $$u\frac{\partial W}{\partial r}+w\frac{\partial W}{\partial z}+\frac{bW_{c}}{C_{w}-C_{\infty }}\left(\frac{\partial W}{\partial z}\frac{\partial C}{\partial z}+W\frac{\partial^{2}C}{\partial z^{2}}\right)=D_{W}\left(\frac{\partial^{2}W}{\partial r^{2}}+\frac{1}{r}\frac{\partial W}{\partial r\;}+\frac{\partial^{2}W}{\partial z^{2}}\right),$$

The apposite boundary conditions are (see [32,36])

$$u=L_{1}\frac{\partial u}{\partial z},\;\;\;v=v_{w}+L_{1}\frac{\partial v}{\partial z},\;\;\;w=0,\;\;\;T=T_{w}+L_{2}\frac{\partial T}{\partial z},$$

(17) $$C=C_{w}+L_{3}\frac{\partial C}{\partial z},\;W=W_{w}+L_{4}\frac{\partial W}{\partial z}\;\mathrm{at}\;z=0$$

(18) $$u\to 0,\;\;\;v\to 0,\;\;\;T\to T_{\infty },C\to C_{\infty },\;W\to W_{\infty }\;\mathrm{as}\;z\to \infty ,$$

where $L_{i}$ (*i* = 1,2,3,4) are the slip coefficients; $v_{w}=r\Omega$; and subscripts w and ∞ denote the disk surface and ambient state, respectively.

Now consider the following transformations ([26,32,42]):

$$\xi =\frac{z}{r}\sqrt{Re},\;\;f^{\prime }\left(\xi \right)=\frac{u}{r\Omega },\;\;\;g\left(\xi \right)=\frac{v}{r\Omega },\;\;\;\;f\left(\xi \right)=\frac{w}{-\sqrt{2\Omega \nu }},$$

(19) $$P\left(\xi \right)=\frac{p_{\infty }-p}{\Omega \mu },\;\;\;\theta \left(\xi \right)=\frac{T-T_{\infty }}{T_{w}-T_{\infty }},\;\;\;\phi \left(\xi \right)=\frac{C-C_{\infty }}{C_{w}-C_{\infty }},\;\;\;\Psi \left(\xi \right)=\frac{W-W_{\infty }}{W_{w}-W_{\infty }}$$

where $\xi ,\;f^{\prime }\left(\xi \right),\;g\left(\xi \right),\;f\left(\xi \right),\;P,\phi ,\;\Psi \left(\xi \right),$ and θ are the dimensionless similarity variable, radial velocity, tangential velocity, axial velocity, pressure, nanoparticle concentration, microorganism distribution, and temperature, respectively.

Plugging Equation (19) into (10)–(18), we obtain the self-similar equations:

(20) $$2f^{\prime \prime \prime }+2ff^{\prime \prime }-f^{\prime 2}+g^{2}-\frac{M}{1+m^{2}}\left(f^{\prime }-mg\right)=0,$$

(21) $$2g^{\prime \prime }+2fg^{\prime }-2f^{\prime }g-\frac{M}{1+m^{2}}\left(g+mf^{\prime }\right)=0,$$

(22) $$P^{\prime }+ff^{\prime }+f^{\prime }=0,$$

(23) $$\frac{3+4R}{3Pr}\theta^{\prime \prime }+f\theta^{\prime }+Nb\theta^{\prime }\phi^{\prime }+Nt\theta^{\prime 2}+Ec\left(f^{\prime \prime 2}+6Re^{-1}f^{\prime 2}\right)=0,$$

(24) $$\phi^{\prime \prime }+PrLef\phi^{\prime }+\frac{Nt}{Nb}\theta^{\prime \prime }=0,$$

(25) $$\;\Psi^{\prime \prime }+Lbf\;\Psi^{\prime }-Pe\left(\phi^{\prime \prime }\left(\Psi +\Xi \right)+\;\Psi^{\prime }\phi^{\prime }\right)=0,$$

Similarly, the boundary conditions (17) and (18) yield

$$f\left(\xi \right)=0,\;\;f^{\prime }\left(\xi \right)=\alpha_{1}f^{\prime \prime }\left(\xi \right),\;\;\;g\left(\xi \right)=1+\alpha_{1}g^{\prime }\left(\xi \right),\;\;\theta \left(\xi \right)=1+\alpha_{2}\theta^{\prime }\left(\xi \right),$$

(26) $$\phi \left(\xi \right)=1+\alpha_{3}\phi^{\prime }\left(\xi \right),\;\Psi \left(\xi \right)=1+\alpha_{4}\Psi^{\prime }\left(\xi \right)\;\mathrm{at}\;\xi =0,$$

(27) $$f^{\prime }\left(\xi \right)\to 0,\;\;\;\;g\left(\xi \right)\to 0,\;\;\;\;\;\theta \left(\xi \right)\to 0,\;\phi \left(\xi \right)\to 0,\;\Psi \left(\xi \right)\to 0\;\mathrm{as}\;\xi \to \infty$$

where $M=\frac{\sigma B_{0}^{2}}{\rho \Omega }$ denotes the magnetic parameter, $R=\frac{4\sigma^{*}T_{\infty }^{3}}{kk^{*}}$ denotes the radiation parameter, $Pr=\frac{\mu C_{p}}{k}$ denotes the Prandtl number, $Nb=\frac{{\left(\rho C_{p}\right)}_{np}D_{B}\left(C_{w}-C_{\infty }\right)}{\mu C_{p}}$ denotes the the Brownian motion parameter, $Le=\frac{k}{\rho C_{p}D_{B}}$ denotes the Lewis number, $Nt=\frac{{\left(\rho C_{p}\right)}_{np}D_{T}\left(T_{w}-T_{\infty }\right)}{T_{\infty }\mu C_{p}}$ denotes the thermophoresis number, $Lb=\frac{\nu }{D_{B}}$ denotes the bioconvection Lewis number, $Pe=\frac{bW_{c}}{D_{W}}$ denotes the Peclet number, $\Xi =\frac{C_{\infty }}{\left(C_{w}-C_{\infty }\right)}$ denotes the nanoparticle concentration ratio, $Ec=\frac{v_{w}^{2}}{C_{p}\left(T_{w}-T_{\infty }\right)}$ denotes the Eckert number, $\alpha_{1}=L_{1}\sqrt{\frac{2\Omega }{\nu }}$ denotes the velocity slip parameter, $\alpha_{2}=L_{2}\sqrt{\frac{2\Omega }{\nu }}$ denotes the thermal slip parameter, $\alpha_{3}=L_{3}\sqrt{\frac{2\Omega }{\nu }}$ denotes the solutal slip parameter, and $\alpha_{4}=L_{4}\sqrt{\frac{2\Omega }{\nu }}$ denotes the microorganism slip parameter.

The shear stress on the disk surface along the tangential and radial directions is given by:

(28) $$\tau_{\varphi }={\left[\mu \left(\frac{\partial v}{\partial z\;}+\frac{1}{z}\frac{\partial w}{\partial \varphi }\right)\right]}_{z=0}$$

(29) $$\tau_{r}={\left[\mu \left(\frac{\partial u}{\partial z\;}+\frac{\partial w}{\partial r}\right)\right]}_{z=0}$$

The total heat flux, nanoparticle mass flux, and microorganism mass flux on the disk surface are defined as:

(30) $$q_{w}={\left[q_{r}-k\frac{\partial T}{\partial z\;}\right]}_{z=0}$$

(31) $$q_{m}={\left[-D_{B}\frac{\partial C}{\partial z\;}\right]}_{z=0}$$

(32) $$q_{n}={\left[-D_{W}\frac{\partial W}{\partial z\;}\right]}_{z=0}$$

Now, the coefficients of the wall friction along the tangential and radial directions, the Nusselt number, the Sherwood number, and the local motile number are computed using:

(33) $$C_{g}=\frac{\tau_{\varphi }}{\frac{1}{2}\rho v_{w}^{2}}$$

(34) $$C_{f}=\frac{\tau_{r}}{\frac{1}{2}\rho v_{w}^{2}},$$

(35) $$Nu=\frac{rq_{w}}{k\left(T_{w}-T_{\infty }\right)}$$

(36) $$Sh=\frac{rq_{m}}{D_{B}\left(C_{w}-C_{\infty }\right)},$$

(37) $$Dn=\frac{rq_{n}}{D_{W}\left(W_{w}-W_{\infty }\right)},$$

The self-similar forms of (33)–(37) are given below:

(38) $$Re^{1/2}C_{g}=g^{\prime }\left(0\right),\;Re^{1/2}C_{f}=f^{\prime \prime }\left(0\right),\;\;Re^{-1/2}Nu=-\left(1+\frac{4}{3}R\right)\theta^{\prime }\left(0\right),\;\;$$

(39) $$Re^{-1/2}Sh=-\phi^{\prime }\left(0\right),\;\;\;\;Re^{-1/2}Dn=-\Psi^{\prime }\left(0\right),$$

where $Re=rv_{w}/\Omega$ is the local Reynolds number.

## 3. Numerical Approach

The nonlinear parametrized and normalized governing Equations (20)–(25) subject to (26) and (27) were coupled, but the solutions in closed form were impractical; therefore, they were solved using the shooting method combined with the Runge–Kutta method (see [43] for more details). As a first step, let us introduce

$$\left(Z_{1},\;Z_{2},\;Z_{3},\;Z_{4},Z_{5},\;Z_{6},\;Z_{7},\;Z_{8},Z_{9},Z_{10},Z_{11}\right)=\left(f,f^{\prime },f^{\prime \prime },g,g^{\prime },\theta ,\;\theta^{\prime },\phi ,\phi^{\prime },\Psi ,\Psi^{\prime }\right),$$

to obtain the initial value problem

(40) $$Z_{1}^{\prime }=Z_{2},\to Z_{1}\left(0\right)=0,$$

(41) $$Z_{2}^{\prime }=Z_{3},\to Z_{2}\left(0\right)=\alpha_{1}s_{1},$$

(42) $$Z_{3}^{\prime }=\left(-2Z_{1}Z_{3}+Z_{2}^{2}-Z_{4}^{2}+\frac{M}{1+m^{2}}\left(Z_{2}-mZ_{4}\right)\right)/2,\;Z_{3}\left(0\right)=s_{1}$$

(43) $$Z_{4}^{\prime }=Z_{5},\to Z_{4}\left(0\right)=1+\alpha_{1}s_{2},$$

(44) $$Z_{5}^{\prime }=\left(-2Z_{1}Z_{5}+2Z_{2}Z_{4}+\frac{M}{1+m^{2}}\left(Z_{4}+mZ_{2}\right)\right)/2,Z_{5}\left(0\right)=s_{2}$$

(45) $$Z_{6}^{\prime }=Z_{7}\to Z_{6}\left(0\right)=1+\alpha_{2}s_{3},$$

(46) $$Z_{7}^{\prime }=\left(\frac{-3Pr}{3+4R}\right)\left(Z_{1}Z_{7}+NbZ_{7}Z_{9}+NtZ_{7}^{2}+Ec\left(Z_{3}^{2}+6Re^{-1}Z_{2}^{2}\right)\right),\to Z_{7}\left(0\right)=s_{3},$$

(47) $$Z_{8}^{\prime }=Z_{9},\;Z_{8}\left(0\right)=1+\alpha_{3}s_{4},$$

(48) $$Z_{9}^{\prime }=-PrLeZ_{1}Z_{9}-\frac{Nt}{Nb}Z_{7}\prime \to Z_{9}\left(0\right)=s_{4},$$

(49) $$Z_{10}^{\prime }=Z_{11},\to Z_{10}\left(0\right)=1+\alpha_{4}s_{5},$$

(50) $$Z_{11}^{\prime }=-LbZ_{1}Z_{11}+Pe\left(Z_{9}^{\prime }\left(Z_{10}+\Xi \right)+Z_{9}Z_{11}\right),\to Z_{11}\left(0\right)=s_{5},$$

where $s_{i}$ (i=1,2,3,4,5) are estimated unknowns. The classical Runge–Kutta method was used to solve the above problem with a suitable guess for $s_{i}$’s. Using the Newton–Raphson method, the guesses were refined until the solution satisfied the required accuracy of $10^{-6}$ (see [44,45] for more details). For a validation of the obtained results, we drew a comparison with the available data (Rehman et al. [34] and Hayat et al. [33]); the compared data are presented in Table 1. Our results coincided exactly with those of [33] and [34].

## 4. Results and Discussion

**Figure 2:**
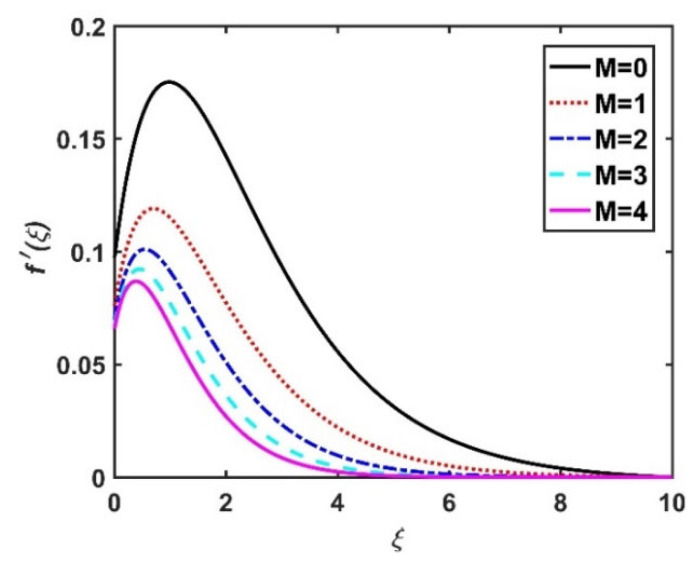
Impact of M on $f\prime \left(\xi \right)$.

**Figure 3:**
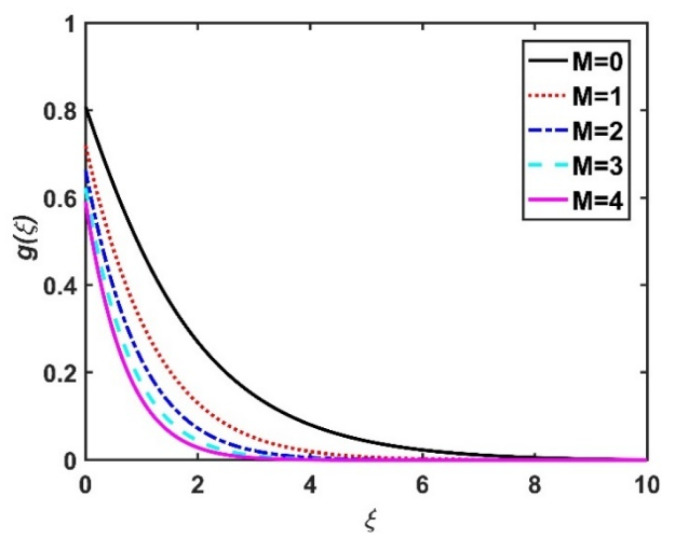
Impact of M on $g\left(\xi \right)$.

**Figure 4:**
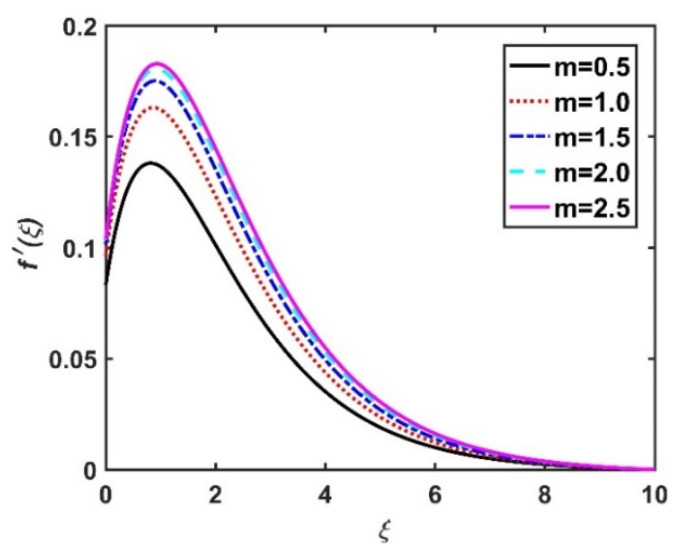
Impact of m on $f\prime \left(\xi \right)$.

**Figure 5:**
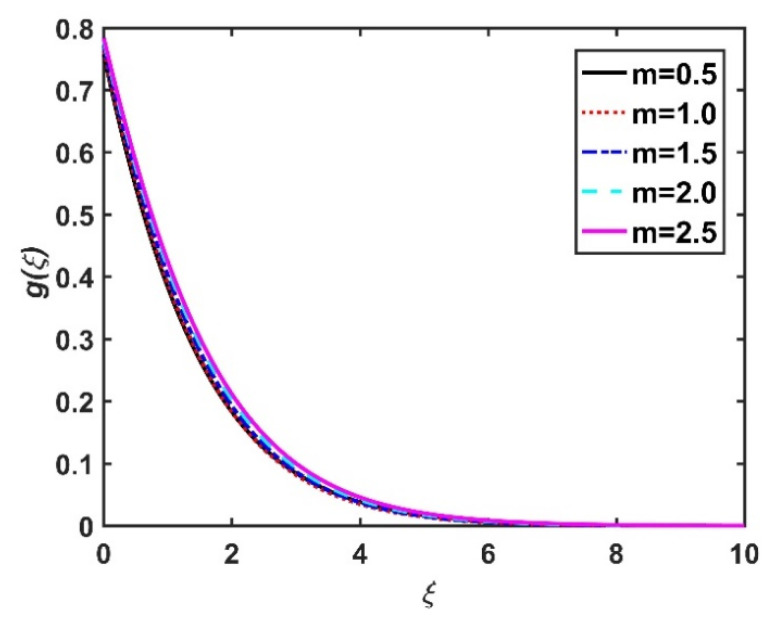
Impact of m on $g\left(\xi \right)$.

**Figure 6:**
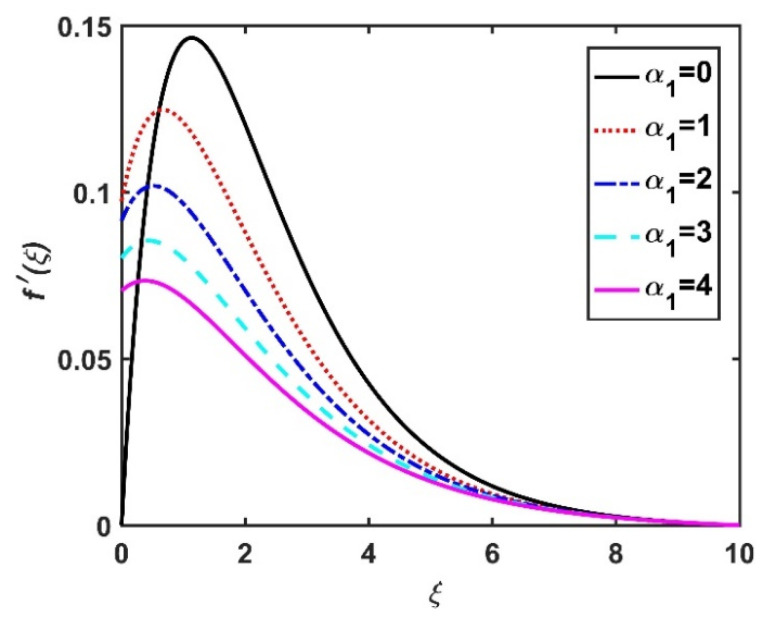
Impact of $\alpha_{1}$ on $f\prime \left(\xi \right)$.

**Figure 7:**
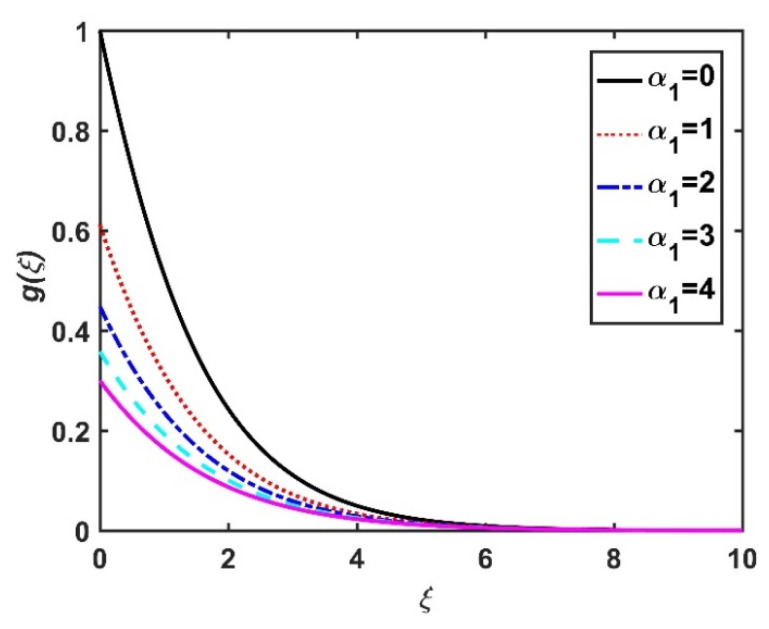
Impact of $\alpha_{1}$ on $g\left(\xi \right)$.

**Figure 8:**
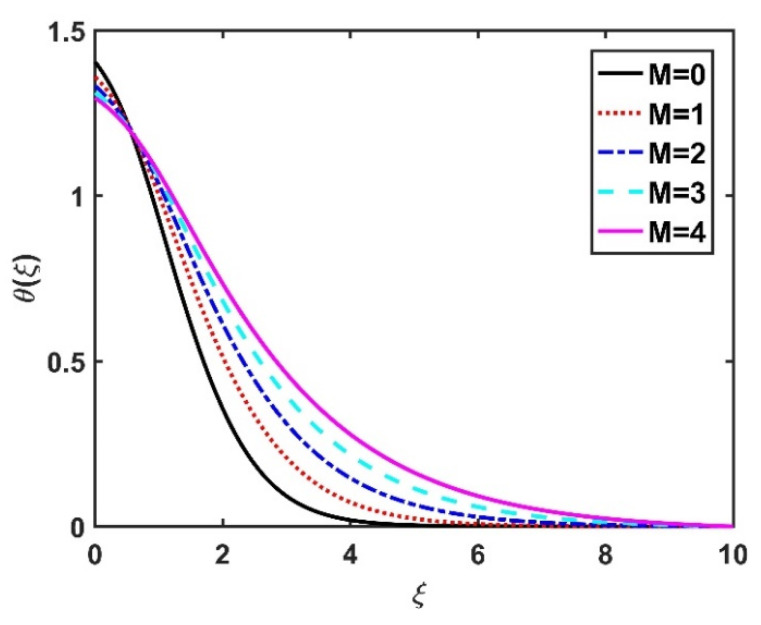
Impact of M on $\theta \left(\xi \right)$.

**Figure 9:**
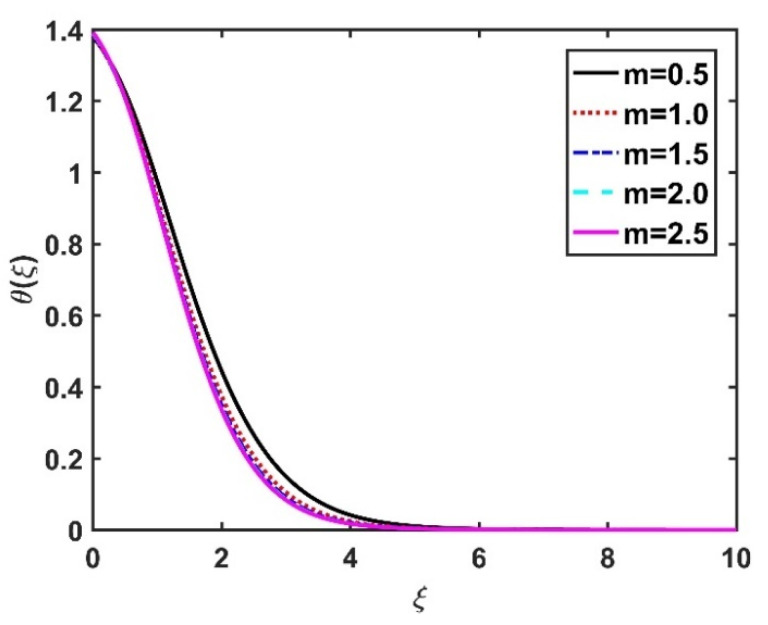
Impact of m on $\theta \left(\xi \right)$.

**Figure 10:**
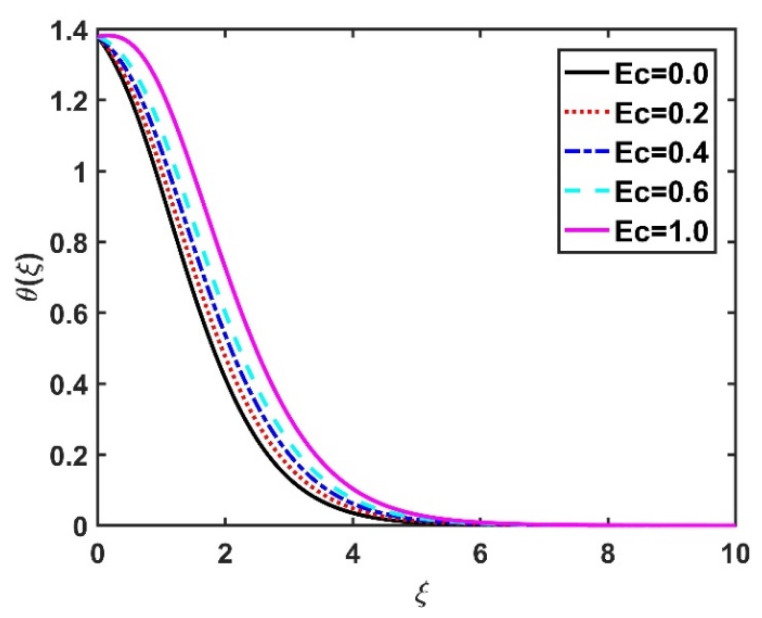
Impact of Ec on $\theta \left(\xi \right)$.

**Figure 11:**
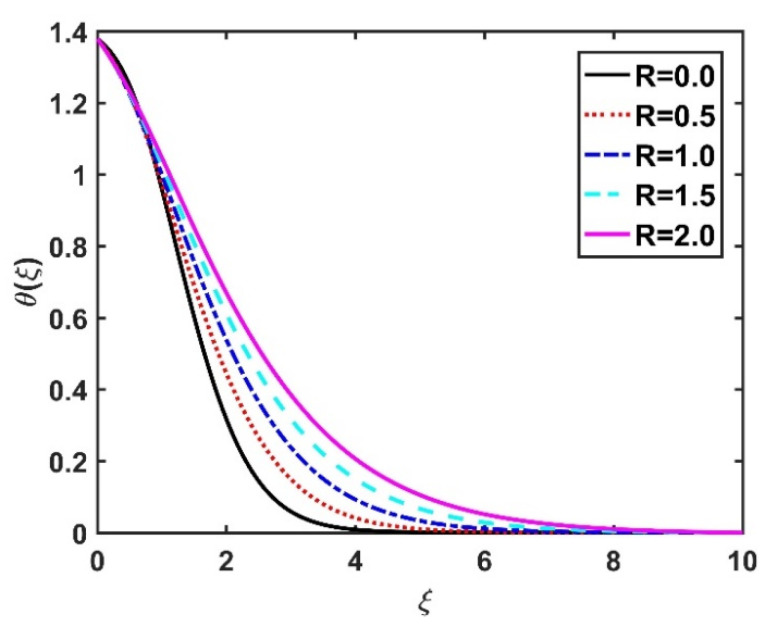
Impact of R on $\theta \left(\xi \right)$.

**Figure 12:**
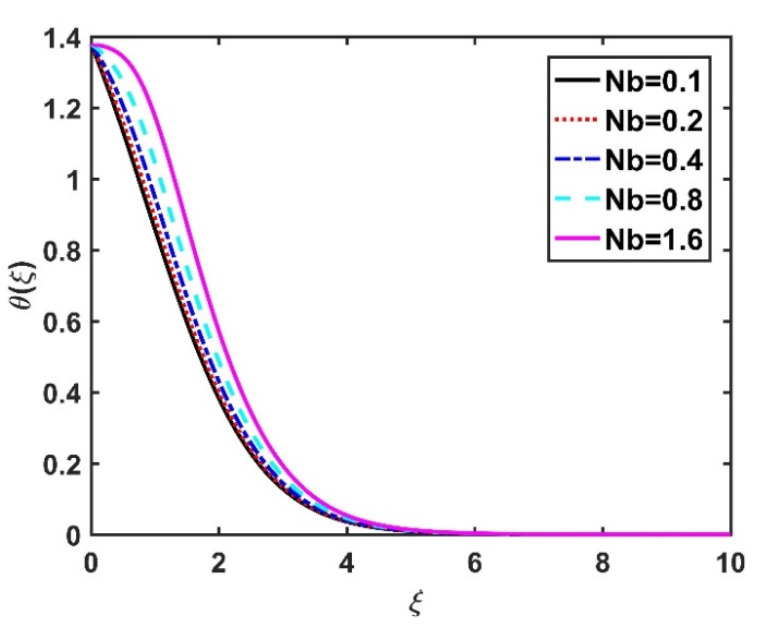
Impact of Nb on $\theta \left(\xi \right)$.

**Figure 13:**
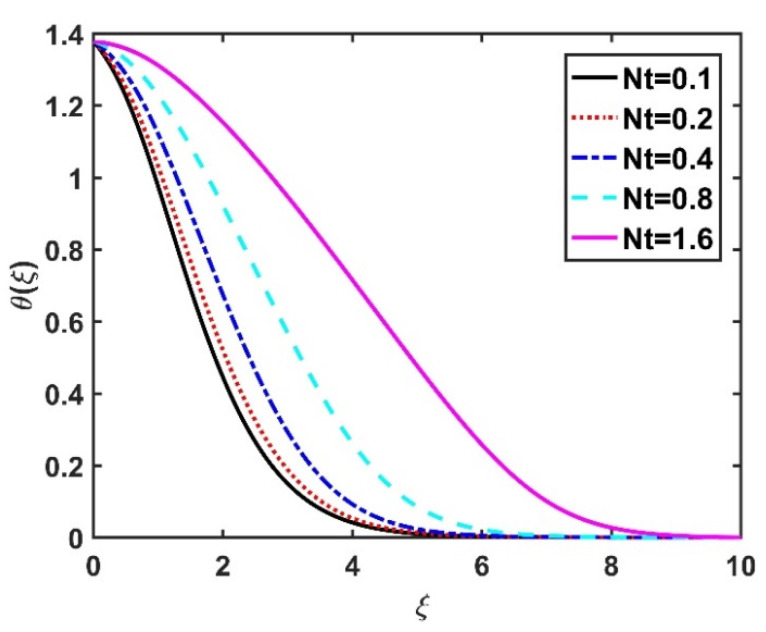
Impact of Nt on $\theta \left(\xi \right)$.

**Figure 14:**
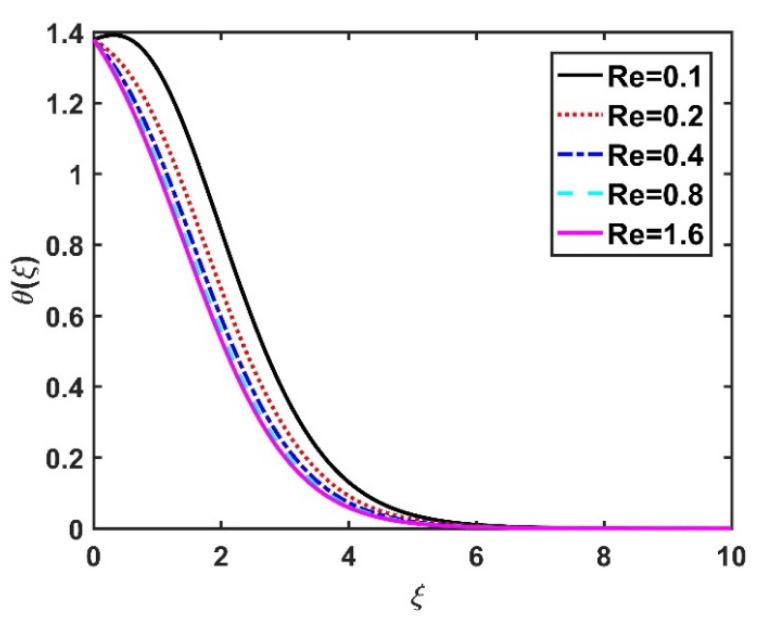
Impact of Re on $\theta \left(\xi \right)$.

**Figure 15:**
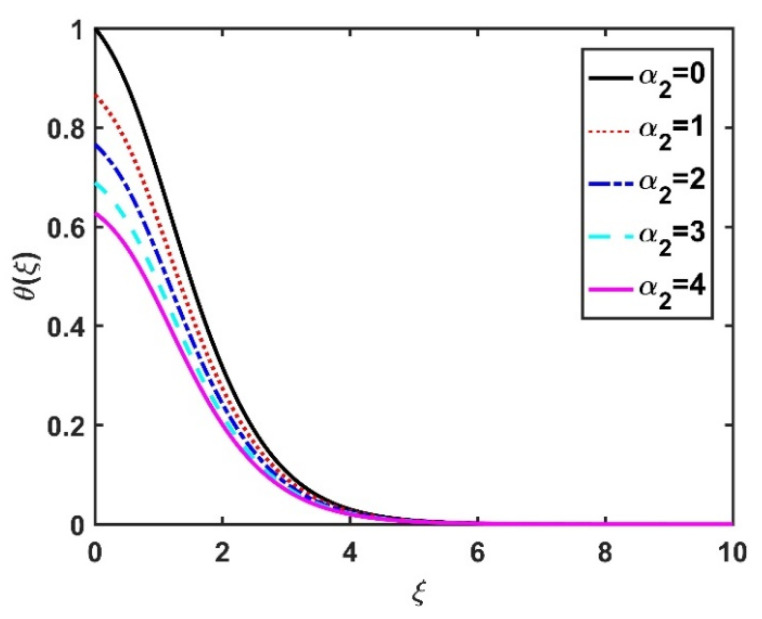
Impact of $\alpha_{2}$ on $\theta \left(\xi \right)$.

**Figure 16:**
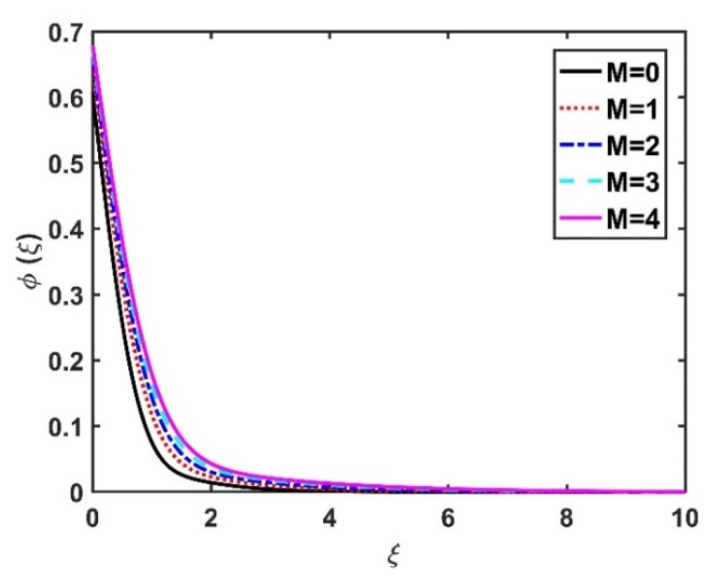
Impact of M on $\phi \left(\xi \right)$.

**Figure 17:**
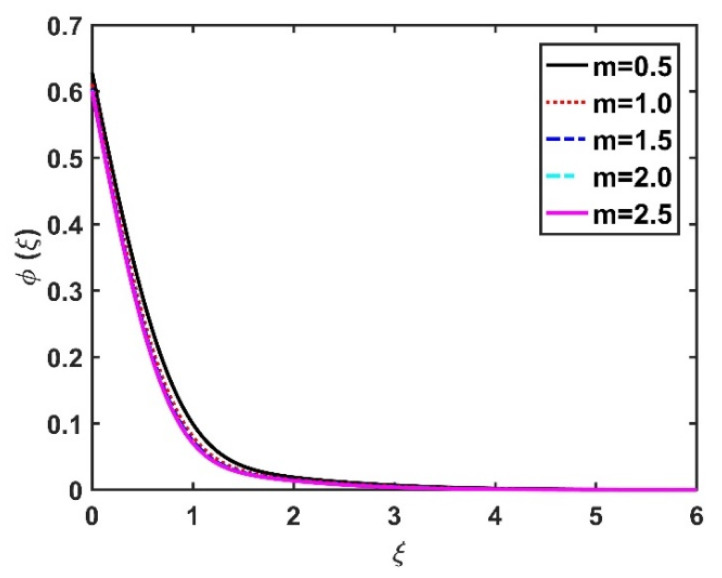
Impact of m on $\phi \left(\xi \right)$.

**Figure 18:**
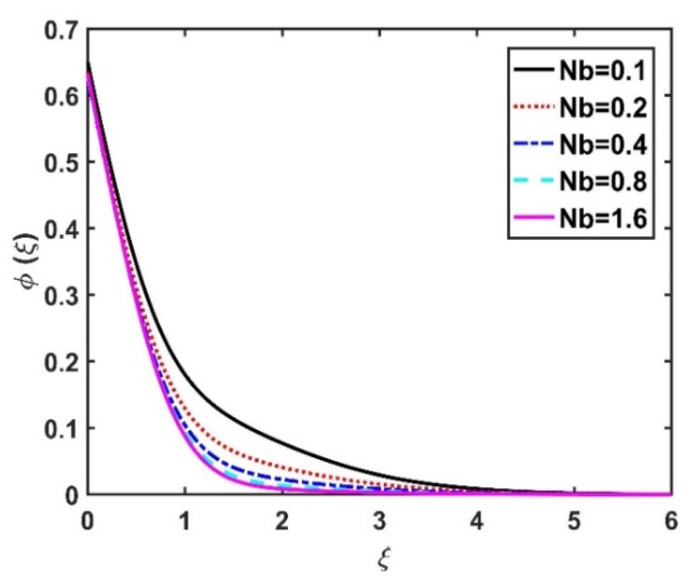
Impact of Nb on $\phi \left(\xi \right)$.

**Figure 19:**
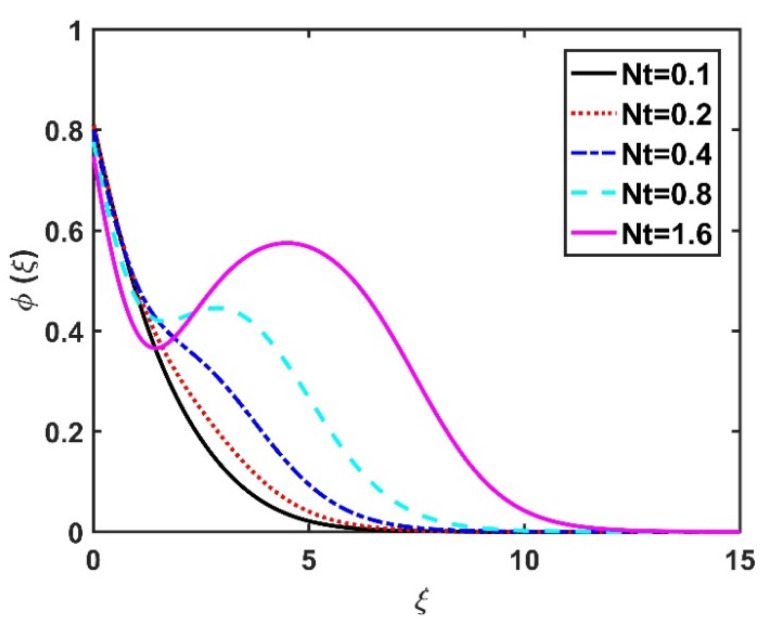
Impact of Nt on $\phi \left(\xi \right)$.

**Figure 20:**
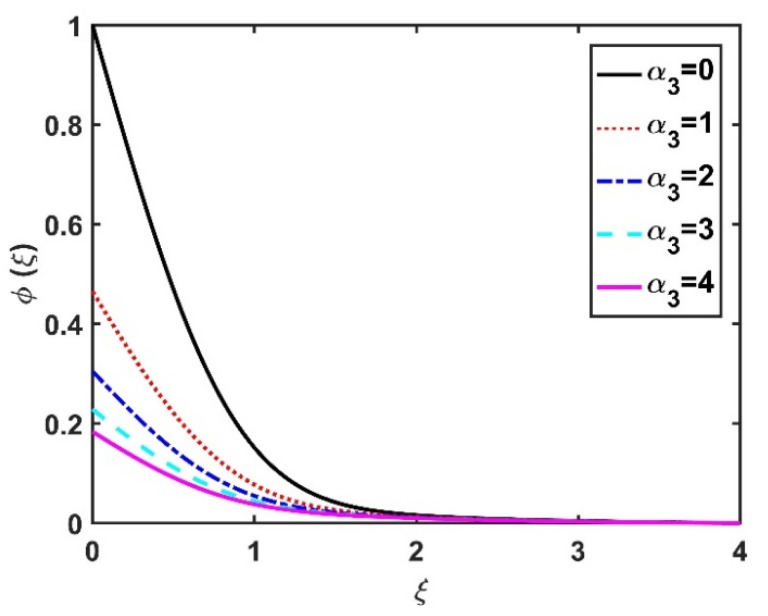
Impact of $\alpha_{3}$ on $\phi \left(\xi \right)$.

**Figure 21:**
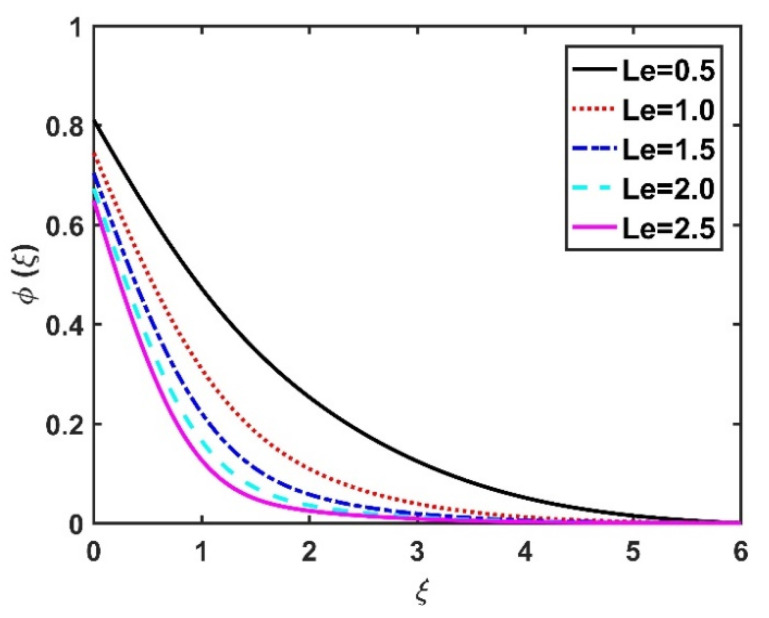
Impact of Le on $\phi \left(\xi \right)$.

**Figure 22:**
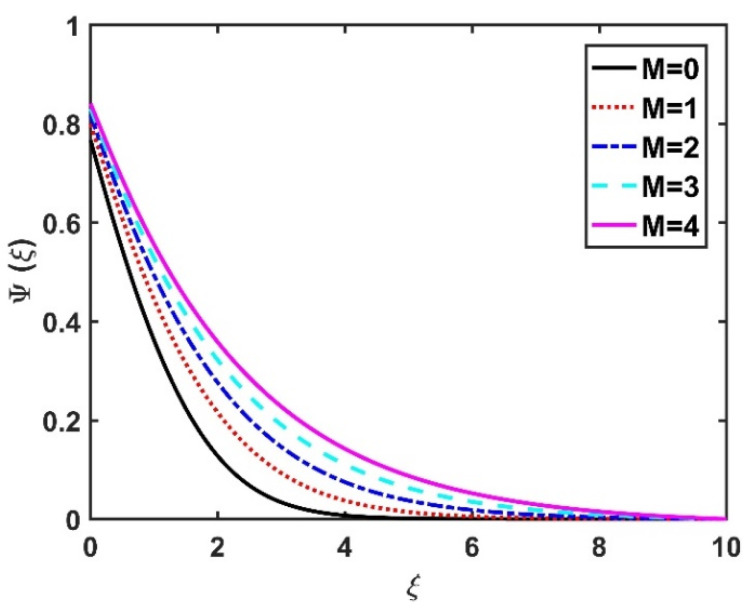
Impact of M on $\Psi \left(\xi \right)$.

**Figure 23:**
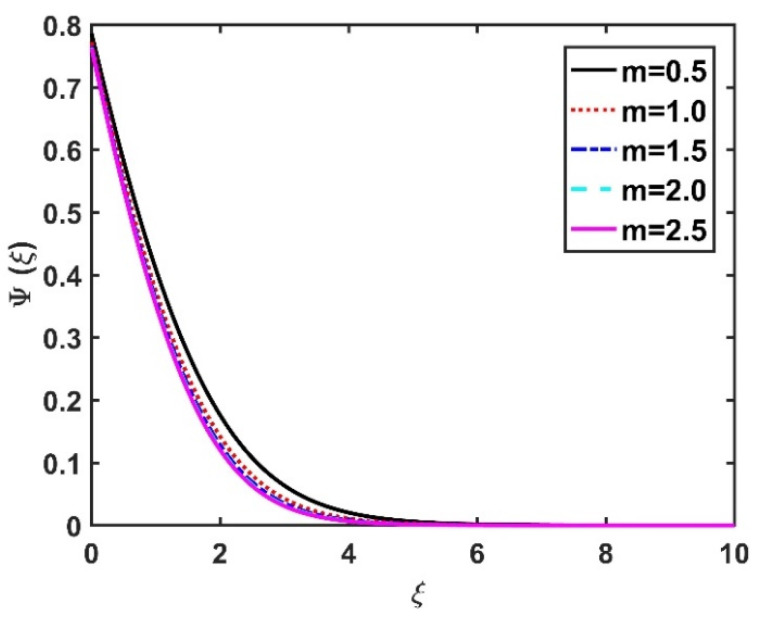
Impact of m on $\Psi \left(\xi \right)$.

**Figure 24:**
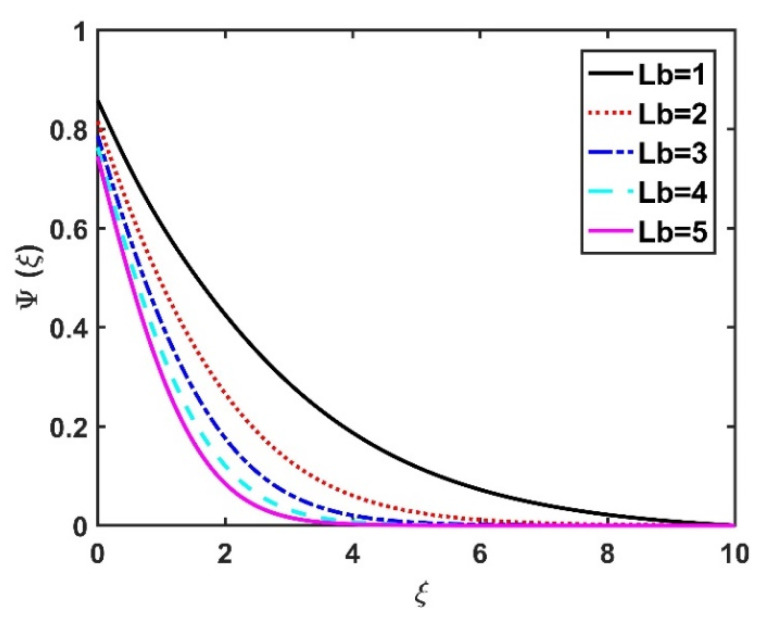
Impact of Lb on $\Psi \left(\xi \right)$.

**Figure 25:**
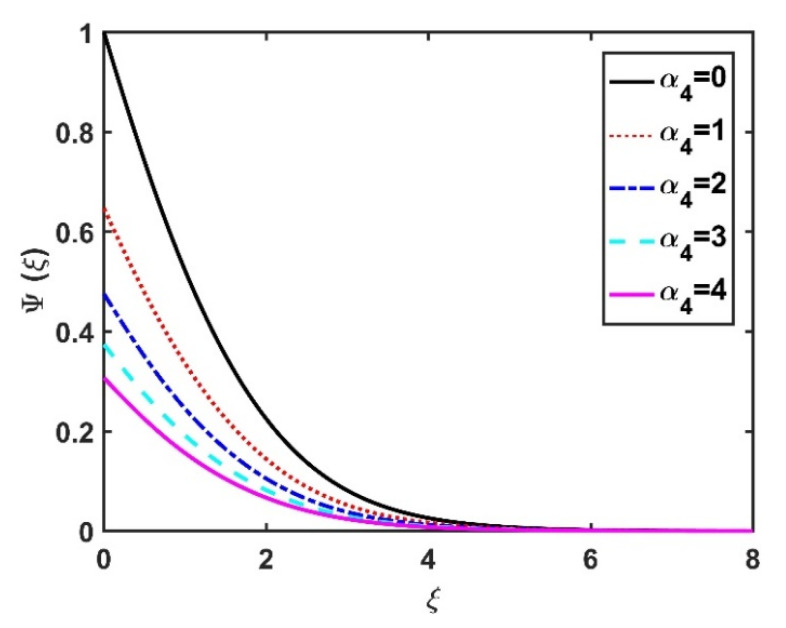
Impact of $\alpha_{4}$ on $\Psi \left(\xi \right)$.

**Figure 26:**
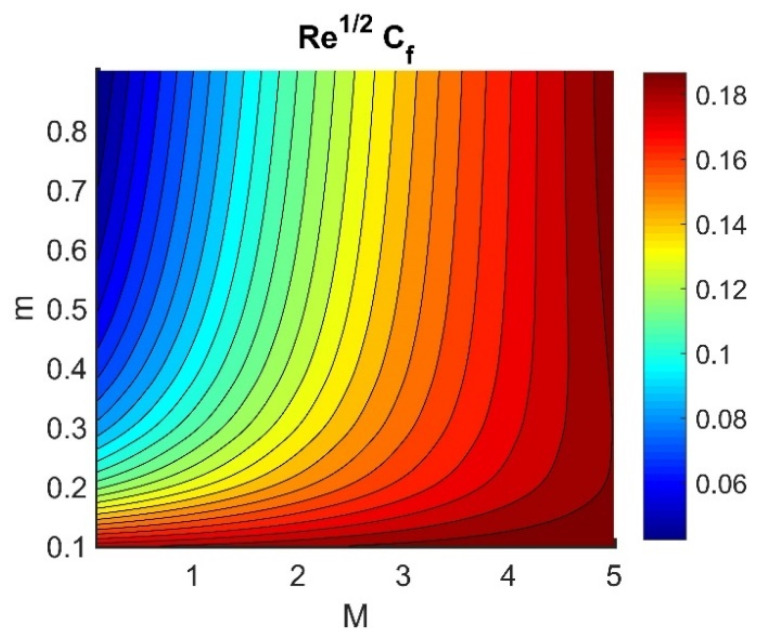
Impact of M and m on Re1/2Cf.

**Figure 27:**
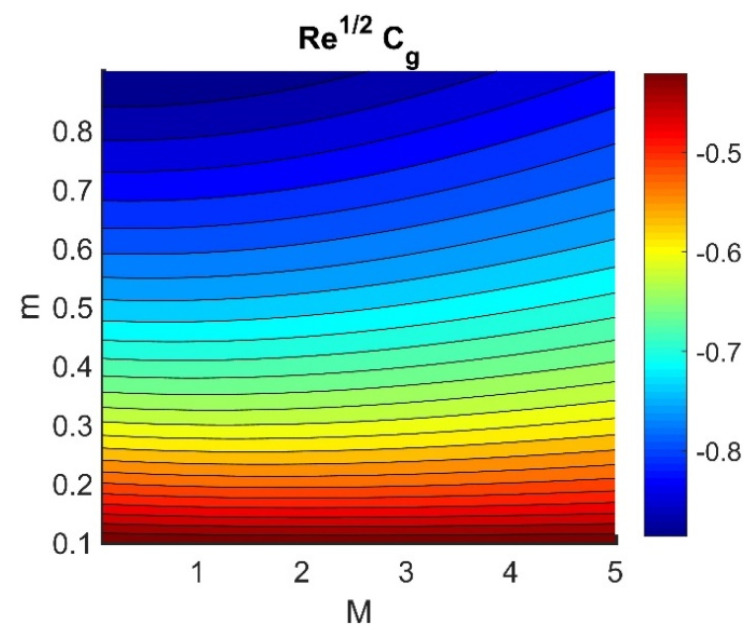
Impact of M and m on Re1/2Cg.

**Figure 28:**
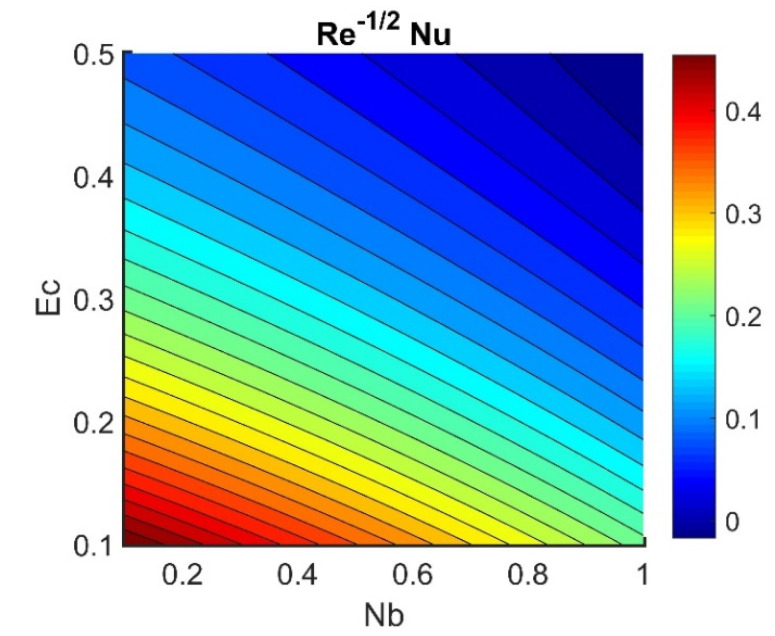
Impact of Ec and Nb on Re−1/2Nu.

**Figure 29:**
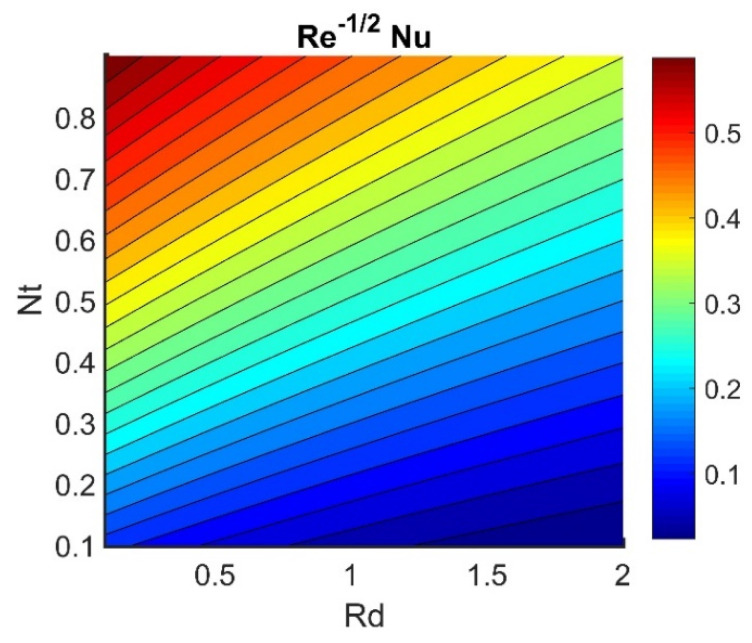
Impact of Nt and Rd on Re−1/2Nu.

**Figure 30:**
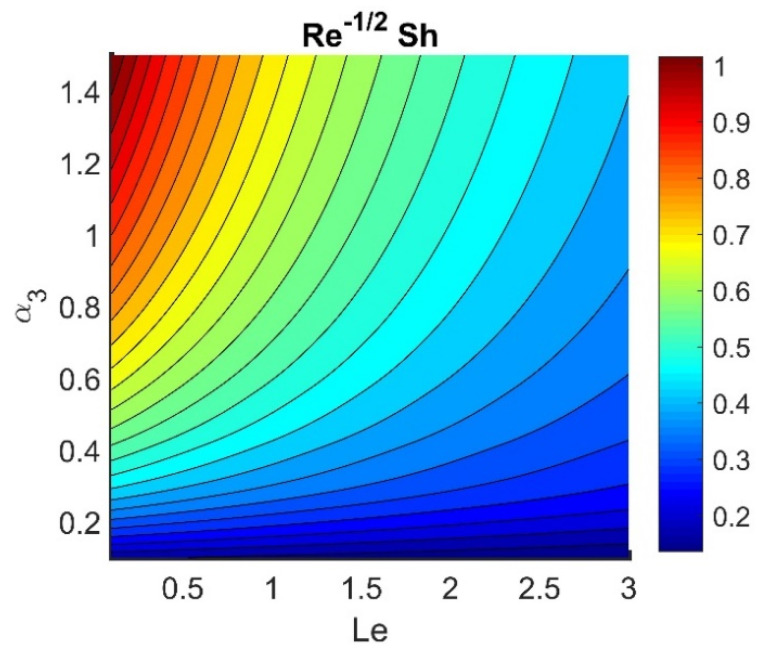
Impact of $Le\;\mathrm{and}\;\alpha_{3}$ on Re−1/2Sh.

**Figure 31:**
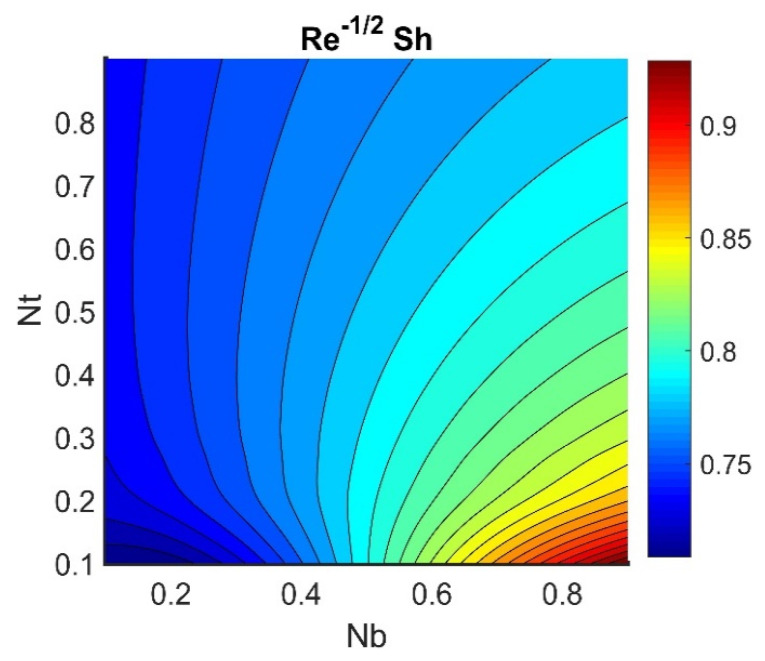
Impact of Nb and Nt on Re−1/2Sh.

In this section, we analyze the physical effects of the magnetic field (M), Hall current (m), velocity slip ($\alpha_{1}$), viscous dissipation (Ec), Rosseland radiation (R), Brownian motion (Nb), thermophoresis (Nt), Reynolds number (Re), thermal slip ($\alpha_{2}$), nanoparticle solute slip ($\alpha_{3}$), Lewis number (Le), motile microorganism slip ($\alpha_{4}$), and bioconvection Lewis number (Lb) on the radial velocity ($f\prime \left(\xi \right)$), tangential velocity ($g\left(\xi \right)$), temperature ($\theta \left(\xi \right)$), nanoparticle concentration ($\phi \left(\xi \right)$), and motile microorganism distribution ($\Psi \left(\xi \right)$) through the graphical representations presented in Figure 2, Figure 3, Figure 4, Figure 5, Figure 6, Figure 7, Figure 8, Figure 9, Figure 10, Figure 11, Figure 12, Figure 13, Figure 14, Figure 15, Figure 16, Figure 17, Figure 18, Figure 19, Figure 20, Figure 21, Figure 22, Figure 23, Figure 24 and Figure 25. Furthermore, the radial wall-friction coefficient (Re1/2Cf), tangential wall-friction coefficient (Re1/2Cg), Nusselt number (Re−1/2Nu), Sherwood number (Re−1/2Sh), and motile microorganism number (Re−1/2Dn) are computed and presented in Figure 26, Figure 27, Figure 28, Figure 29, Figure 30 and Figure 31 and Table 2 and Table 3. In the simulations, unless indicated otherwise, we set $Pr=6,\;Ha=m=R=\alpha_{1}=\alpha_{2}=\alpha_{3}=\alpha_{4}=Nb=0.5,\;Ec=Nt=Pe=0.1,Re=1,\;Le=Lb=3$, and Ξ=0.2.

### 4.1. Velocity Profiles ($f^{\prime }\left(\xi \right),g\left(\xi \right)$)

Figure 2 and Figure 3 show the effect of the magnetic number M on the radial velocity ($f^{\prime }\left(\xi \right)$) and tangential velocity ($g\left(\xi \right)$), respectively. Both velocities were reduced under a stronger magnetic field, because the higher the M value, the greater the Lorentz force (typically a repellent force). The Lorentz force opposes fluid motion on the surface of a disk, and hence the fluid motion slowed down in both the radial and tangential directions. Therefore, both $f^{\prime }\left(\xi \right)$ and $g\left(\xi \right)$ decreased as M increased. Here, M=0 corresponds to the absence of a Lorentz force, at which point the velocities were highest over the entire surface of the disk. The effect of m on $f^{\prime }\left(\xi \right)$ and $g\left(\xi \right)$ is depicted in Figure 4 and Figure 5, respectively. Note that a stronger Hall current facilitates the movement of the fluid, so the velocities increased with m. The Hall current had an increased effect on the radial velocity compared to the velocity in other directions. Figure 6 and Figure 7 present the impact of $\alpha_{1}$ on $f^{\prime }\left(\xi \right)$ and $g\left(\xi \right)$. Here, $\alpha_{1}=0$ corresponds to the no-slip velocity conditions. The radial velocity on the disk surface was greater under the slip velocity conditions, whereas the tangential velocity component on the disk surface was smaller under the slip conditions. Both velocity components ($f^{\prime }\left(\xi \right)$ and $g\left(\xi \right)$) diminished with the increase in the values of $\alpha_{1}$. Physically, the slip conditions on the disk serve as a repellent agent that reduces the velocity.

### 4.2. Temperature Profile ($\theta \left(\xi \right)$)

Figure 8 and Figure 9 illustrate the effect of M and m on $\theta \left(\xi \right)$. In the absence of a magnetic field, the temperature $\theta \left(\xi \right)$ on the disk surface was lower. Increasing the M value led to a higher temperature $\theta \left(\xi \right)$, while the opposite trend was observed for increasing the m value. Figure 10 shows that the temperature $\theta \left(\xi \right)$ increased steadily as the Eckert numbers increased. Physically, the kinetic energy resulting from viscous heating acts as a heat-generating agent that improves the temperature $\theta \left(\xi \right)$. Figure 11 shows that increasing the radiation number led to a significant improvement in the temperature $\theta \left(\xi \right)$ due to the electromagnetic waves exerted by the solar radiation. Figure 11 demonstrates that the solution converged when R=0. The impacts of Nb and Nt on $\theta \left(\xi \right)$ are depicted in Figure 12 and Figure 13. Brownian motion creates additional thermal energy due to the accidental motion of nanoparticles, which improves the thermal distribution $\theta \left(\xi \right)$. Likewise, the heat migration of the nanoparticles acts as a heat-generating agent, causing an increase in $\theta \left(\xi \right)$. The Nt had more of a pronounced effect than the Nb on the $\theta \left(\xi \right)$. Larger Reynolds numbers (Re) correspond to a weaker viscous force, and such a weaker viscous force reduced the $\theta \left(\xi \right)$. In Figure 15, $\alpha_{2}=0$. represents conditions without thermal slip, where $\theta \left(\xi \right)$ had the value 1, corresponding to isothermal conditions. For a larger $\alpha_{2}$, the temperature $\theta \left(\xi \right)$ dropped on the disk surface and decreased throughout the fluid region as $\alpha_{2}$ increased.

### 4.3. Nanoparticle Concentration Profile ($\phi \left(\xi \right)$)

The effects of the Hall current and magnetic field on $\phi \left(\xi \right)$ were contradictory, as shown in Figure 16 and Figure 17. That is, $\phi \left(\xi \right)$ rose at the M values decreased and the values of m increased. The increase in the Brownian number reduced $\phi \left(\xi \right),$ as shown in Figure 18. The greater the strength of Brownian motion, the lower the concentration gradient of the nanoparticles; therefore, $\phi \left(\xi \right)$ was reduced. The impact of Nt on $\phi \left(\xi \right)$ is reported in Figure 19, which shows that the $\phi \left(\xi \right)$ increased pointedly with rising Nt values. The higher the Nt value, the higher the nanoparticle concentration gradient, which caused $\phi \left(\xi \right)$ to increase unevenly. In Figure 20, $\alpha_{3}=0$ does not imply slip conditions in the solute, and therefore the $\phi \left(\xi \right)$ increased on the disk surface. Furthermore, increasing the $\alpha_{3}$ values pointedly diminished the nanoparticle concentration profile. Figure 21 shows that $\phi \left(\xi \right)$ declined with an increase in the Lewis number. Physically, the larger the Le, the weaker the diffusivity of the solute, thus reducing the $\phi \left(\xi \right)\;\mathrm{value}$.

### 4.4. Motile Microorganism Profile ($\Psi \left(\xi \right)$)

Figure 22 and Figure 23 illustrate the motile microorganism distribution $\Psi \left(\xi \right)$ with respect to the magnetic number $\left(M\right)$ and the Hall number $\left(m\right)$, respectively. The $\Psi \left(\xi \right)\;\mathrm{profile}$ decreased with an increase in the Hall factor $\left(m\right)$, while it improved with a rise in the M value. Physically, the Lorentz force facilitates the increased diffusivity of microorganisms and, thereby, amplifies the $\Psi \left(\xi \right)\;\mathrm{profile}$. The higher the bioconvection Lewis number, the weaker the diffusivity of the bioconvection; therefore, for larger Lb values, the $\Psi \left(\xi \right)$ profile became smaller, as seen in Figure 24 and Figure 25, which display the effects of $\alpha_{4}$ on the $\Psi \left(\xi \right)$ profile. The microorganism profile decreased under slip conditions.

### 4.5. Physical Quantities ($Re^{1/2}C_{f}$, $Re^{1/2}C_{g}$, $Re^{-1/2}Nu$, $Re^{-1/2}Sh$, and $Re^{-1/2}Dn$)

Figure 26 shows the contours of Re1/2Cf plotted against m and M. Increasing the m value reduced the Re1/2Cf, while the effect of M was almost invariant for different values of m. A lower value of M and a higher value of m produced the lowest radial shear stress on the disk wall. Figure 27 demonstrates that Re1/2Cg did not vary with respect to M for different values of m. Lower values of Ec and Nb achieved the maximum Re−1/2Nu value (see Figure 28). Figure 29 shows that Re−1/2Nu decreased with increased radiation, while it rose in conjunction with Nt. Therefore, to achieve a maximum Re−1/2Nu, the Nt value must be as high as possible and the R value must be low. The effects of $\alpha_{3}$ and Le on Re−1/2Sh conflicted (see Figure 30). The non-linear effects of Nb and Nt on Re−1/2Sh are illustrated in Figure 31.

Table 2 presents the behavior of Re1/2Cf, Re1/2Cg, Re−1/2Nu, Re−1/2Sh, and Re−1/2Dn for different values of $m,\;\alpha_{1}$, and M. Under no-slip velocity conditions, Re1/2Cf, Re1/2Cg, Re−1/2Nu, and Re−1/2Sh were higher than under velocity slip conditions, while this result was reversed for Re−1/2Dn. Both Re1/2Cf and Re1/2Cg increased in conjunction with m, while the opposite was true for M. Re−1/2Nu, Re−1/2Sh, and Re−1/2Dn increased with m, while the opposite was true for M. Table 3 presents the behavior of Re−1/2Nu, Re−1/2Sh, and Re−1/2Dn for different values of $R,\;\alpha_{2},\alpha_{3},$ and $\alpha_{4}$. Re−1/2Nu increased as the values of R and $\alpha_{3}$ increased, while this outcome was reversed for $\alpha_{2}$ and $\alpha_{4}$. Re−1/2Sh and Re−1/2Dn diminished as $R,\;\alpha_{2},\alpha_{3}$, and $\alpha_{4}\;\mathrm{increased}$.

## 5. Concluding Remarks

Three-dimensional bioconvection viscous dissipating nanofluid flow on a spinning disk was investigated, along with the Hall current, magnetic field, radiative heat transfer, and multiple slip effects. The framework of the Buongiorno nanofluid model was employed. The system of non-linear PDEs was simplified into dimensionless ODEs through a similarity scheme, then solved using the shooting technique (see [46,47,48,49,50]). The main results of the above analysis were:

- The radial velocity diminished with an increase in the magnetic field and rose with an increase in the Hall current.
- The velocities declined as a result of higher velocity slip parameters.
- The temperature field was improved under higher magnetic number, Eckert number, and radiation parameter values.
- The thermophoresis number had a greater impact on the heat field compared to the Brownian number.
- Multiple slip conditions reduced the transport fields.
- The frictional coefficient of the wall in the radial direction was reduced by an increase the Hall current.
- The heat transfer rate was reduced by an increase in the Brownian motion number, while an increase in thermal radiation elevated the heat transfer rate.
- An increase in Brownian motion and thermophoresis reduced the rate of heat transfer.

## Figures and Tables

**Table 1:** The values of $Re^{-1/2}Nu$ for different values of M and $\alpha_{1}$ when $Rd=m=\alpha_{2}=\alpha_{3}=Ec=0$ in the absence of microorganisms.

M	$\alpha_{1}$	Hayat et al. [33]	Rehman et al. [34]	Our Results
0	0.7	0.30494	0.3050	0.304948
0.7	0.7	0.24421	0.2443	0.244214
1.4	0.7	0.17566	0.1757	0.175661
0.3	0.2	0.32655	0.3266	0.326552
0.3	0.5	0.30360	0.3036	0.303607
0.3	0.8	0.28715	0.2872	0.287155

**Table 2:** The values of Re1/2Cf, Re1/2Cg, Re−1/2Nu, Re−1/2Sh, and Re−1/2Dn for different values of $m,\;\alpha_{1}$, and M when $Pr=6,\;R=\alpha_{2}=\alpha_{3}=\alpha_{4}=Nb=0.5,\;Ec=Nt=Pe=0.1,Re=1,\;Le=Lb=3$, and Ξ=0.2.

$\alpha_{1}$	m	M	$Re^{1/2}C_{f}$	$Re^{1/2}C_{g}$	$Re^{-1/2}Nu$	$Re^{-1/2}Sh$	$Re^{-1/2}Dn$
0	0	0.5	0.27223285	−0.60016174	0.18411756	0.61217292	0.35891396
	0.5		0.34804091	−0.60760591	0.20678466	0.66211221	0.40193257
	1		0.38657686	−0.57472358	0.21535335	0.68590441	0.42344578
	0.5	0	0.36000722	−0.43512123	0.21557962	0.68362256	0.42656590
		1	0.35017124	−0.75136693	0.19808066	0.64951373	0.38490344
		2	0.34939527	−0.98614427	0.18346595	0.63623923	0.36227622
1	0	0.5	0.06907399	−0.37685871	0.19043449	0.65499791	0.35472070
	0.5		0.09729511	−0.38822687	0.23499319	0.73620866	0.41653888
	1		0.11529524	−0.38145137	0.25654230	0.77752075	0.44911090
	0.5	0	0.12206753	−0.31990335	0.26542362	0.79444265	0.46550481
		1	0.08479902	−0.43742667	0.21112374	0.69834925	0.38281983
		2	0.07294442	−0.50408669	0.17797708	0.65135755	0.33905929

**Table 3:** The values of Re−1/2Nu, Re−1/2Sh, and Re−1/2Dn for different values of $R,\;\alpha_{2},\alpha_{3}$, and $\alpha_{4}$ when $Pr=6,\;\alpha_{1}=R=Ha=m=Nb=0.5,\;Ec=Nt=Pe=0.1,Re=1,\;Le=Lb=3$, and Ξ=0.2.

R	$\alpha_{2}$	$\alpha_{3}$	$\alpha_{4}$	$Re^{-1/2}Nu$	$Re^{-1/2}Sh$	$Re^{-1/2}Dn$
0	0.5	0.5	0.5	0.09536398	0.74735703	0.42765668
1				0.37593681	0.73549955	0.42662967
2				0.61025676	0.73202599	0.42630997
0.5	0	0.5	0.5	0.25909380	0.74013993	0.42704913
	1			0.22314167	0.73889796	0.42692132
	2			0.19518753	0.73798530	0.42682639
0.5	0.5	0	0.5	0.13514058	1.17259288	0.45258266
		1		0.30584709	0.53909050	0.41493130
		2		0.37956428	0.34920648	0.40338675
0.5	0.5	0.5	0	0.23995186	0.73946890	0.53998522
			1	0.23995170	0.73946891	0.35308806
			2	0.23995170	0.73946888	0.26230148

## Data Availability

Not applicable.

## Nomenclature

*u, v, w*	Velocity components $\left[m\cdot s^{-1}\right]$
r, φ,z	$\mathrm{Space}\;\mathrm{coordinates}\;\left[m\right]$
$B_{0}$	Magnetic field strength $\left[N\cdot m^{-1}\cdot A^{-1}\right]$
${\left(\rho C\right)}_{p}$	Nanoparticle specific heat $\left[J\cdot kg^{-3}\cdot K^{-1}\right]$
$\alpha_{1}$	Velocity slip parameter
$\alpha_{2}$	Temperature slip parameter
$\alpha_{3}$	Concentration slip parameter
$\alpha_{4}$	Microorganism slip parameter
Pr	Prandtl parameter
Lb	Bioconvection Lewis number
*R*	Radiation parameter
Le	Lewis number
Pe	Peclet parameter
M	Magnetic parameter
Nu	Nusselt number
$C_{f},\;C_{g}$	Friction coefficients
Sh	Sherwood number
Dn	Motile number
*b*	Chemotaxis constant $\left[m\right]$
$W_{\infty }$	Ambient microorganisms
$T_{\infty }$	Ambient temperature $\left[K\right]$
$C_{\infty }$	Ambient concentration
$D_{B}$	Brownian diffusivity $\left[m^{2}\cdot s^{-1}\right]$
$D_{w}$	Microorganism diffusivity $\left[m^{2}\cdot s^{-1}\right]$
ν	Kinematic viscosity $\left[m^{2}\cdot s^{-1}\right]$
Ec	Eckert number
σ	Electrical conductivity $\left[S\cdot m^{-1}\right]$
$W_{c}$	Speed of cell swimming $\left[m\cdot s^{-1}\right]$
$D_{T}$	Thermophoresis diffusivity $\left[m^{2}\cdot s^{-1}\right]$
ρ	Fluid density $\left[kg\cdot m^{-3}\right]$
$C_{w}$	Concentration at wall
$W_{w}$	Microorganisms at wall
$T_{w}$	Temperature at wall $\left[K\right]$
Nt	Thermophoresis parameter
Nb	Brownian motion parameter

## References

[1] Abu-Nada E. (2008). Application of nanofluids for heat transfer enhancement of separated flows encountered in a backward facing step. Int. J. Heat Fluid Flow.

[2] Choi S.U., Eastman J.A. (1995). Enhancing Thermal Conductivity of Fluids with Nanoparticles.

[3] Buongiorno J. (2006). Convective transport in nanofluids. J. Heat Transf..

[4] Kuznetsov A.V., Nield D.A. (2010). Natural convective boundary-layer flow of a nanofluid past a vertical plate. Int. J. Therm. Sci..

[5] Nield D.A., Kuznetsov A.V. (2009). The Cheng–Minkowycz problem for natural convective boundary-layer flow in a porous medium saturated by a nanofluid. Int. J. Heat Mass Transf..

[6] Wakif A., Zaydan M., Alshomrani A.S., Muhammad T., Sehaqui R. (2022). New insights into the dynamics of alumina-(60% ethylene glycol+ 40% water) over an isothermal stretching sheet using a renovated Buongiorno’s approach: A numerical GDQLLM analysis. Int. Commun. Heat Mass Transf..

[7] Rasheed H.U., Khan W., Khan I., Alshammari N., Hamadneh N. (2022). Numerical computation of 3D Brownian motion of thin film nanofluid flow of convective heat transfer over a stretchable rotating surface. Sci. Rep..

[8] Gumber P., Yaseen M., Rawat S.K., Kumar M. (2022). Heat transfer in micropolar hybrid nanofluid flow past a vertical plate in the presence of thermal radiation and suction/injection effects. Partial Differ. Equ. Appl. Math..

[9] Mahanthesh B. (2021). Flow and heat transport of nanomaterial with quadratic radiative heat flux and aggregation kinematics of nanoparticles. Int. Commun. Heat Mass Transf..

[10] Areekara S., Mackolil J., Mahanthesh B., Mathew A., Rana P. (2022). A study on nanoliquid flow with irregular heat source and realistic boundary conditions: A modified Buongiorno model for biomedical applications. ZAMM-J. Appl. Math. Mech./Z. Für Angew. Math. Und Mech..

[11] Farooq U., Waqas H., Imran M., Albakri A., Muhammad T. (2022). Numerical investigation for melting heat transport of nanofluids due to stretching surface with Cattaneo-Christov thermal model. Alex. Eng. J..

[12] Dawar A., Wakif A., Saeed A., Shah Z., Muhammad T., Kumam P. (2022). Significance of Lorentz forces on Jeffrey nanofluid flows over a convectively heated flat surface featured by multiple velocity slips and dual stretching constraint: A homotopy analysis approach. J. Comput. Des. Eng..

[13] Nazeer M., Khan M.I., Khan S.U., Saleem A., Muhammad T., Shah S.I. (2022). Assessment of heat and mass transfer characteristics in Poiseuille flow of non-Newtonian nanofluid in a porous channel with convectively heated lower wall. Chin. J. Phys..

[14] Mishra S.R., Sun T.C., Rout B.C., Khan M.I., Alaoui M.K., Khan S.U. (2022). Control of dusty nanofluid due to the interaction on dust particles in a conducting medium: Numerical investigation. Alex. Eng. J..

[15] Li P., Abbasi A., El-Zahar E.R., Farooq W., Hussain Z., Khan S.U., Khan M.I., Farooq S., Malik M., Wang F. (2022). Hall effects and viscous dissipation applications in peristaltic transport of Jeffrey nanofluid due to wave frame. Colloid Interface Sci. Commun..

[16] Kuznetsov A.V. (2011). Nanofluid bioconvection in water-based suspensions containing nanoparticles and oxytactic microorganisms: Oscillatory instability. Nanoscale Res. Lett..

[17] Sampath Kumar P.B., Gireesha B.J., Mahanthesh B., Chamkha A.J. (2019). Thermal analysis of nanofluid flow containing gyrotactic microorganisms in bioconvection and second-order slip with convective condition. J. Therm. Anal. Calorim..

[18] Chu Y.M., Shankaralingappa B.M., Gireesha B.J., Alzahrani F., Khan M.I., Khan S.U. (2022). Combined impact of Cattaneo-Christov double diffusion and radiative heat flux on bio-convective flow of Maxwell liquid configured by a stretched nano-material surface. Appl. Math. Comput..

[19] Ayodeji F., Tope A., Pele O. (2020). Magneto-hydrodynamics (MHD) Bioconvection nanofluid slip flow over a stretching sheet with thermophoresis, viscous dissipation and brownian motion. Mach. Learn. Res..

[20] Khan M., Salahuddin T., Malik M.Y., Alqarni M.S., Alqahtani A.M. (2020). Numerical modeling and analysis of bioconvection on MHD flow due to an upper paraboloid surface of revolution. Phys. A Stat. Mech. Its Appl..

[21] Shehzad S.A., Mushtaq T., Abbas Z., Rauf A., Khan S.U., Tlili I. (2020). Dynamics of bioconvection flow of micropolar nanoparticles with Cattaneo-Christov expressions. Appl. Math. Mech..

[22] Khan M.I., Haq F., Khan S.A., Hayat T., Khan M.I. (2020). Development of thixotropic nanomaterial in fluid flow with gyrotactic microorganisms, activation energy, mixed convection. Comput. Methods Programs Biomed..

[23] Ferdows M., Zaimi K., Rashad A.M., Nabwey H.A. (2020). MHD bioconvection flow and heat transfer of nanofluid through an exponentially stretchable sheet. Symmetry.

[24] Waqas H., Khan S.U., Bhatti M.M., Imran M. (2020). Significance of bioconvection in chemical reactive flow of magnetized Carreau–Yasuda nanofluid with thermal radiation and second-order slip. J. Therm. Anal. Calorim..

[25] Muhammad T., Waqas H., Mahanthesh B. (2021). Computational analysis of bioconvection in magnetized flow of thixotropic nanofluid with gyrotactic microorganisms. Math. Fluid Mech. Adv. Convect. Instab. Incompressible Fluid Flow.

[26] Von Karman T. (1939). Classical problem of rotating disk. Transfer ASME.

[27] Benton E.R. (1966). On the flow due to a rotating disk. J. Fluid Mech..

[28] Turkyilmazoglu M. (2011). Heat and mass transfer on the MHD fluid flow due to a porous rotating disk with hall current and variable properties. J. Heat Transf..

[29] Sheikholeslami M., Hatami M., Ganji D.D. (2014). Nanofluid flow and heat transfer in a rotating system in the presence of a magnetic field. J. Mol. Liq..

[30] Turkyilmazoglu M. (2014). Nanofluid flow and heat transfer due to a rotating disk. Comput. Fluids.

[31] Hayat T., Rashid M., Imtiaz M., Alsaedi A. (2015). Magnetohydrodynamic (MHD) flow of Cu-water nanofluid due to a rotating disk with partial slip. AIP Adv..

[32] Abdel-Wahed M., Akl M. (2016). Effect of hall current on MHD flow of a nanofluid with variable properties due to a rotating disk with viscous dissipation and nonlinear thermal radiation. AIP Adv..

[33] Hayat T., Muhammad T., Shehzad S.A., Alsaedi A. (2017). On magnetohydrodynamic flow of nanofluid due to a rotating disk with slip effect: A numerical study. Comput. Methods Appl. Mech. Eng..

[34] Rehman K.U., Malik M.Y., Zahri M., Tahir M. (2018). Numerical analysis of MHD Casson Navier’s slip nanofluid flow yield by rigid rotating disk. Results Phys..

[35] Shehzad S.A., Reddy M.G., Rauf A., Abbas Z. (2020). Bioconvection of Maxwell nanofluid under the influence of double diffusive Cattaneo–Christov theories over isolated rotating disk. Phys. Scr..

[36] Waqas H., Naseem R., Muhammad T., Farooq U. (2021). Bioconvection flow of Casson nanofluid by rotating disk with motile microorganisms. J. Mater. Res. Technol..

[37] Jawad M., Saeed A., Khan A., Islam S. (2021). MHD bioconvection Darcy-Forchheimer flow of Casson nanofluid over a rotating disk with entropy optimization. Heat Transf..

[38] Shehzad S.A., Abbas Z., Rauf A., Mushtaq T. (2020). Effectiveness of Hall current and thermophysical properties in compressible flow of viscous fluid thorough spinning oscillatory disk. Int. Commun. Heat Mass Transf..

[39] Khan M., Ali W., Ahmed J. (2020). A hybrid approach to study the influence of Hall current in radiative nanofluid flow over a rotating disk. Appl. Nanosci..

[40] Bég O.A., Kabir M.N., Uddin M.J., Izani Md Ismail A., Alginahi Y.M. (2021). Numerical investigation of Von Karman swirling bioconvective nanofluid transport from a rotating disk in a porous medium with Stefan blowing and anisotropic slip effects. Proc. Inst. Mech. Eng. Part C J. Mech. Eng. Sci..

[41] Rana P., Mackolil J., Mahanthesh B., Muhammad T. (2022). Cattaneo-Christov Theory to model heat flux effect on nanoliquid slip flow over a spinning disk with nanoparticle aggregation and Hall current. Waves Random Complex Media.

[42] Rana P., Mahanthesh B., Thriveni K., Muhammad T. (2022). Significance of aggregation of nanoparticles, activation energy, and Hall current to enhance the heat transfer phenomena in a nanofluid: A sensitivity analysis. Waves Random Complex Media.

[43] Ha S.N. (2001). A nonlinear shooting method for two-point boundary value problems. Comput. Math. Appl..

[44] Mathews J.H., Fink K.D. (2004). Numerical Methods Using MATLAB.

[45] Beers K.J., Beers K.J. (2007). Numerical Methods for Chemical Engineering: Applications in Matlab.

[46] Pal D., Mandal G. (2017). Effects of Hall current on magnetohydrodynamic heat transfer of nanofluids over a non-linear stretching/shrinking sheet. Int. J. Appl. Comput. Math..

[47] Pal D., Mandal G. (2019). Magnetohydrodynamic heat and mass transfer of Sisko nanofluid past a stretching sheet with nonlinear thermal radiation and convective boundary condition. J. Nanofluids.

[48] Mandal G., Pal D. (2021). Entropy generation analysis of radiated magnetohydrodynamic flow of carbon nanotubes nanofluids with variable conductivity and diffusivity subjected to chemical reaction. J. Nanofluids.

[49] Pal D., Mandal G. (2021). Magnetohydrodynamic nonlinear thermal radiative heat transfer of nanofluids over a flat plate in a porous medium in existence of variable thermal conductivity and chemical reaction. Int. J. Ambient Energy.

[50] Mandal G., Pal D. (2022). Entropy generation analysis of magnetohydrodynamic Darcy-Forchheimer Williamson hybrid nanofluid flow through porous medium with nonlinear thermal radiation. Spec. Top. Rev. Porous Media Int. J..

